# What is important to people with dementia living at home? A set of core outcome items for use in the evaluation of non-pharmacological community-based health and social care interventions

**DOI:** 10.1093/ageing/afaa015

**Published:** 2020-02-17

**Authors:** Siobhan T Reilly, Andrew J E Harding, Hazel Morbey, Faraz Ahmed, Paula R Williamson, Caroline Swarbrick, Iracema Leroi, Linda Davies, David Reeves, Fiona Holland, Mark Hann, John Keady

**Affiliations:** 1 Faculty of Health and Medicine, Division of Health Research, Lancaster University, Lancaster, UK; 2 Clinical Trials Research Centre, University of Liverpool, Liverpool, UK; 3 Medical Research Council North West Hub for Trials Methodology Research, Liverpool, UK; 4 School of Medicine, Trinity College Dublin, Dublin, Ireland; 5 Division of Population Health, Health Services Research and Primary Care, School of Health Sciences, University of Manchester, Manchester, UK; 6 NIHR School for Primary Care Research, School of Health Sciences, University of Manchester, Manchester, UK; 7 Division of Nursing, Midwifery and Social Work, University of Manchester, Manchester, UK; 8 Greater Manchester Mental Health NHS Foundation Trust, Manchester, UK

**Keywords:** core outcome set, dementia, non-pharmacological, psychosocial, outcomes, older people

## Abstract

**Objectives:**

inconsistency in outcome measurement in dementia care trials impedes the comparisons of effectiveness between trials. The key aim of this study is to establish an agreed standardised core outcome set (COS) for use when evaluating non-pharmacological health and social care interventions for people with dementia living at home.

**Method:**

we used a mixed-methods research design, including substantive qualitative research with five key stakeholders groups. We consulted with people living with dementia for many aspects of this research. We applied a modified two-round 54 item Delphi approach to attain consensus on core outcomes. The COS was finalised in a face-to-face consensus meeting in 2018.

**Results:**

of the 288 who completed round 1 (21 people living with dementia, 58 care partners, 137 relevant health and social care professionals, 60 researchers, 12 policy makers), 246 completed round 2 (85% response rate). Twenty participants attended the consensus meeting. We reached consensus for the inclusion of 13 outcome items.

**Conclusion:**

we identified 13 outcome items which are considered core; many relate to social health. Providing there are adequate measures, measuring these core outcome items will enhance comparisons for effectiveness making trial evidence more useful. The items will provide commissioners and service planners with information on what types of interventions are most likely to be valued highly by people living with dementia.

**Trial registration:**

The study is registered on the COMET initiative database.

## Key points

- There is a high variability in outcomes and measurement instruments used in non-pharmacological dementia trials; this makes it difficult to compare for effectiveness.
- Core outcome sets address this problem by gaining consensus on outcomes that should be measured in all trials.
- We have gained consensus from key stakeholders on 13 core outcome items considered core for all non-pharmacological dementia trials.
- A key strength of this work is the involvement of people living with dementia in the research design process and as participants.
- Thirteen outcomes items were identified as core; these are what people value in order to live well with dementia.

## Introduction

Dementia interventions and outcomes continue to be central pillars of dementia strategies and policies at global and national levels. For example, the World Health Organisation Global action plan on public health has seven cross cutting themes—one of which is a call ‘to develop strategies and interventions for dementia care that are person-centred, cost-effective, sustainable and affordable, and take public health principles and cultural aspects into account’ [1]. Similarly, a central recommendation from a taskforce of leading UK clinicians and researchers in dementia, UK funders of dementia research, people with dementia and carer representatives, is to identify priority areas for dementia research ‘…to understand how to achieve the best outcomes possible’ [2]. A critical precursor to identifying effective interventions, or understanding how to achieve the best outcomes, is to first identify which outcomes are regarded as important by stakeholders [3]—including people living with dementia.

The authors of a recent systematic review of outcomes of importance to patients with mild cognitive impairment or Alzheimer’s disease, their caregivers, and health-care professionals conclude that trials rarely include many important outcomes. The researchers conclude that including outcomes that people with lived experience value ‘…could help ensure that successful treatments or evaluation of the quality of care is better focused on aspects of Alzheimer’s Disease most important to the people affected by it’ [4]. This underlines the widely held view that dementia care research is a field in which the quality of evidence needs to be stronger [2, 3]. Currently, many dementia-related systematic reviews and clinical guidelines highlight the high degree of variation in outcomes and measures used in existing trials of non-pharmacological health and social care community-based interventions for people living with dementia [5–8]. This variation reduces the quality, robustness and generalisability of the existing evidence and a lack of consistency in outcomes leads to heterogeneity and reporting biases [9, 10] contributing to research waste [11]. Comparisons across studies for effectiveness are obstructed making the interpretation of results, synthesis of evidence and meta-analysis difficult [12].

A high proportion of the 850,000 people estimated to be living with dementia in the UK [13] reside at home and in their everyday neighbourhood [14]. It is crucial to increase the quality of evidence in non-pharmacological health and social care community-based interventions for people living with dementia. The scope of these interventions is broad and includes: psycho-social interventions; psychological interventions; social programmes (e.g. a memory café); case management/care coordination interventions; assistive technology; arts-based activities; and educational programmes.

One way to attain consensus on outcomes of importance is to develop a core outcome set (COS). A COS constitutes outcomes to be measured and reported as a minimum across all relevant effectiveness trials linked to the health or social care area. The use of COS better enables comparisons for effectiveness, increases the quality of evidence and permits an optimal synthesis of evidence [15]. Spearheaded by the Core Outcome Measure in Effectiveness Trials (COMET) initiative (http://www.comet-initiative.org/), the impetus to use COS in research and trials that focus on the effectiveness of interventions is increasing. Key research funders such as the National Institute for Health Research in the UK and Horizon2020 in the EU encourage applicants for research funding to use COS.

Currently, six consensus exercises make recommendations for outcomes of non-pharmacological interventions in both research and care [16–21]. These consensus exercises tend to recommend broad domains that have sub-categories or constructs, for example quality of life and a focus on activities of daily living features in all six consensus recommendations. Cognition features in four of the six recommendations. Neuropsychiatric and behavioural domains both feature in three.

None of the six existing consensus recommendations (see Appendix 1 in Supplementary data) meet all of the standards of COS development [22], including the systematic use of rigorous consensus methods or involvement of key stakeholders. Indeed, these six prior consensus exercises place more emphasis and weight upon the participation of professionals relative to people living with dementia.

The central aim of this research is to use systematic, rigorous and established consensus methods and to involve key stakeholders (particularly people living with dementia [23]) in the research process to develop a COS that can be used when evaluating non-pharmacological health and social care community-based interventions for people living with dementia at home. We defined ‘home’ as where someone usually lives in the community, which includes sheltered or extra care housing, but does not include residential or nursing home care. In this paper, we focus on ‘what to measure’ and present the findings from a modified Delphi approach and consensus meeting used to finalise the agreed COS. The reporting in this paper adheres to the COS reporting standards recommended [24].

## Method

We have drawn on COMET guidance to develop the protocol for this study. There are three phases to this study.

The initial phase of the study involved extracting outcome items of importance from 35 face-to-face and telephone interviews and four focus groups with 55 participants (people living with dementia *n* = 17; care partners *n* = 18; health and social care professionals *n* = 15; policy makers *n* = 4; researchers *n* = 1) and a literature review of existing research, key reviews and policy documents. We initially produced a long list of 170 outcome items which we distilled into 54 outcome items in four conceptual categories (friendly neighbourhood and home, independence, self-managing dementia symptoms, quality of life) [25]. The second phase includes a modified Delphi approach and a consensus meeting and is outlined in this paper.

### Modified Delphi approach

The Delphi method is a structured method for reaching consensus, where participants complete sequential rounds of anonymised surveys. We undertook substantive qualitative work alongside people living with dementia and care partners in their capacity as co-researchers to develop a modified Delphi approach, including the use of a three point as opposed to a nine point scale, and this is outlined in detail elsewhere [23]. We collected the two rounds of Delphi data between November 2017 and February 2018. In round 1, participants were asked to rate the importance of each outcome on a three point scale (Not particularly important, Important, Very important). Round 2 involved participants reviewing round 1 scores (including the scores of other participant groups). Participants were able to review and change their responses [26].

We administered each survey verbally to people living with dementia. In round 2, the participants views were verbally contrasted with the views of health and social care professionals. This was a key modification to the traditional online Delphi approach. A discussion then took place on whether the participant wished to keep their score or change their response for round 2. This paper-based and researcher administration of the modified Delphi survey to the key stakeholder group drew heavily on qualitative methods for planning and in its delivery.

An online survey using DelphiManager (http://www.comet-initiative.org/delphimanager/) was available to other stakeholder groups (care partners, health and social care professionals, policy makers and researchers). Round 1 responses, illustrated in a histogram, were available for respondents receiving the round 2 online survey. All participants, regardless of method of administration, were able to add additional outcomes in round 1.

The consensus criteria adopted were defined as:

- consensus in: 70% or more participants in each stakeholder group scoring the outcome as ‘very important’ and less than 15% participants in each stakeholder group scoring the outcome as ‘not particularly important’;
- consensus out: less than 70% of participants in each stakeholder group scoring as ‘Very important’; and
- no consensus: anything else not included in the other two categories.

The consensus criteria we used differed to the one stated in the protocol; reasons for changing the criteria are presented in Appendix 2 in Supplementary data.

### Consensus meeting

The COS was finalised in an in-person consensus meeting in March 2018 with specialist facilitation from an independent chair (KC). The first part of the consensus meeting sought to discuss and ratify outcomes considered ‘consensus in’ through the Delphi. Utilising focus group methods, participants from each stakeholder group then proceeded to engage in mixed small group discussions on outcomes where no consensus for inclusion was attained through the Delphi approach. The merits of each ‘no consensus’ outcome were discussed in detail. Members of the wider Neighbourhoods and Dementia team (CS and RE) and an independent chair (KC) facilitated the small groups. All participants were asked to independently score each ‘no consensus’ outcome. Eight outcomes at a time were discussed in small groups. All participants scored the outcomes on the paper-based Delphi survey format that was administered to people living with dementia. All participants placed their paper-based slip in an outcome specific ballot box at the end of each series of discussion. To be included in the COS, outcomes required 70% of participants to rate the outcome as ‘Very Important’.

### Recruitment

People with dementia living at home (with capacity) and care partners were recruited from the north-west of England to the Delphi survey and consensus meeting from a variety of community-based settings. Our study protocol provides a full description of the inclusion criteria, recruitment and consent process for all participant groups; we recruited from the UK [26].

### Data analysis

Any outcomes added at the end of round 1 were reviewed by the members of the research team (AH, FA, HM). Both rounds of the modified Delphi approach were analysed using STATA. Round 1 responses were analysed by calculating the percentage of participants scoring each outcome as ‘Very important’, ‘Important’ and ‘Not particularly important’. Corresponding histograms, by stakeholder group, were also produced in STATA and uploaded to DelphiManager at round 2. Analysis for round 2 followed the aforementioned consensus criteria.

Histograms by stakeholder group to visually represent round 2 responses were also produced for the consensus meeting as a tool for use at the facilitator’s discretion to encourage discussion.

Round 2 responses were analysed by calculating the percentage of participants scoring each outcome as ‘Very important’, ‘Important’ and ‘Not particularly important’, and the final consensus criteria were applied to inform the consensus meeting.

## Results

### Modified Delphi survey

Table 1 provides a breakdown of Delphi survey participants. Of the 288 who completed round 1, 246 completed round 2 (85% response rate). Response rates differed across stakeholder groups. For example, 95% of people living with dementia who participated in round 1 completed round 2. The response rates between rounds for the other groups who completed the online survey varied between 80 and 92% (care partners 86%; health and social care professionals 80%; researchers 92%).

At round 1, four outcomes were considered ‘consensus in’ (hygiene and comfort, communication, importance of relationships, meaningful activities). Consensus was not reached for 34 outcomes, while 16 outcomes were considered ‘consensus out’. People living with dementia tended to give a lower percentage score, and at this stage, consensus was often not achieved due to their scores being lower than 70% ‘very important’. Appendix 3 in Supplementary data presents the results from rounds 1 and 2 of the Delphi survey together with those from the consensus meeting.

Ninety-seven additional outcomes were added by participants and reviewed by AH, FA and HM. All of these items were categorised as processes (and therefore were excluded), were not outcomes, or were sufficiently similar to the 54 existing outcomes in the Delphi. No additional new outcomes were added to round 2.

During the completion of round 2, if participants changed their score, they were able to offer a reason (as a free text option/verbally). Overall, 1,463 reasons for a change in score were recorded. For people living with dementia who changed their score, often a particular experience in their day-to-day life led them to value an outcome more or less. However, the vast majority (1,135; 76%) of reasons given came from professional groups (health and social care professionals, policy makers and researchers). Nearly two-thirds (65%) of the reasons for professionals changing their score were to align with the views of people living with dementia. The status of the 54 outcomes after the second round is outlined in Appendix 3 in Supplementary data and was as follows:

- Ten outcomes met the ‘consensus in’ criteria (70% or more of all groups score ‘Very important’ and <15% scored ‘Not particularly important’).
- Twenty outcomes met the ‘consensus out’ criteria (less than 70% of all groups score ‘Very important’).
- Twenty-four outcomes where there was considered to be partial agreement (where the ‘consensus in’ criteria were met in some but not all groups).

### Consensus meeting

Twenty-one participants attended the consensus meeting. There was approximately an equal number of those with lived experiences (people living with dementia and care partners) and those from professional groups (health and social care professionals, policy makers and researchers) (see Table 1). The small groups had 6–7 participants in with a mix of stakeholder background. The histograms produced to visually represent round 2 responses were not needed. The 10 outcomes agreed by the participants as ‘consensus in’ through the Delphi were ratified and no objections were raised for their inclusion in the COS. We chose not to discuss all the ‘consensus out’ outcomes at the consensus meeting as this would have been an overload of information. Discussion and scoring took place to consider the importance of the 24 ‘no consensus’ outcomes; three met the criteria to be considered ‘consensus in’. Thirteen outcomes were included in the final COS (Table 2).

**Table 1:** Delphi and consensus participants in each round of the Delphi and consensus meeting

	Round 1 (November 2017–January 2018)	Round 2 (February 2018)	Consensus meeting (March 2018)
People living with dementia	21^a^	20	6
Carer partners	58	50	5 (including 1 person living with dementia and 1 policy maker)
Health and social care professionals	137 (18 also identified as a researcher and 4 as a policy maker)	109	6
Researchers	60 (14 also identified as health and social care professional)	55	3
Policy makers	12 (2 also identified as a researcher and 2 as a health and social care professional)	12	1 (also a carer)
Total	288	246	21

^a^Characteristics of participants living with dementia.

Sex: males (*n* = 13); females (*n* = 8). One male dropped out in round 2.

Age: 50–54 years *n* = 1; 55–59 years *n* = 3; 60–64 years; *n* = 1; 65–69 years *n* = 4; 70–74 years *n* = 4; 75–79 years; *n* = 3; 80–84 years *n* = 3; 85–89 years *n* = 1; 90+ years *n* = 1.

Other diagnosis: angina or long-term heart problem *n* = 5, arthritis or long-term joint problem *n* = 3, asthma or long-term chest problem *n* = 2, blindness or severe visual impairment *n* = 2, deafness or severe hearing impairment *n* = 4, epilepsy *n* = 2, high blood pressure *n* = 2, kidney or liver disease *n* = 2, long-term back problem *n* = 4, long-term mental health problem *n* = 1, long-term neurological disorder *n* = 2 and type 2 diabetes *n* = 1.

Living arrangements: *n* = 21 lived with their spouse/partner.

**Table 2:** Final Core Outcome Set (COS)

Domain	Outcome item	Lay outcome term (if applicable)	Lay description of outcome item	Percentage of people living with dementia that rated the item as very important in round 2
Friendly neighbourhood and home	1. Importance of relationships	–	Continuing good relationships with people who are important to you	95%
	2. Communication	–	Being able to communicate with others	85%
	3. Feeling safe and secure	–	Feeling safe and secure at home	70%
	4. Feeling valued and respected by others^*^	–	Feeling valued and respected by others	50%
Independence	5. Meaningful activities	–	Being able to do things that you enjoy and want to keep doing	90%
Self-managing dementia symptoms	6. Apathy/indifference	Losing interest	Keeping interested in things you like	80%
	7. Alertness	–	Being aware of your surroundings indoors and outdoors	80%
	8. Understanding time and place^*^	Knowing where you are	Being able to find your way around a familiar place	60%
Quality of life	9. Hygiene and comfort	Personal hygiene & cleanliness	Being as clean and comfortable as you would like	80%
	10. Stability	Falls	Not falling at home or when out and about	79%
	11. Vision and hearing	–	Being able to see, hear and understand	70%
	12. A sense of who you are	–	Feeling able to keep your identity	70%
	13. Having a laugh^*^	–	Feeling able to have a laugh with other people	70%

^*^denotes the outcome item was added to the COS at the consensus meeting. Care partners, health and social care professionals, policy makers and researchers had access to the lay outcome term (or ‘outcome item’ if lay outcome term not applicable) and lay description of the outcome item during the online Delphi survey. People living with dementia had access to the lay description of the outcome only during the Delphi survey. During the consensus meeting, all participants had access to the lay description of the outcome only during the Delphi survey.

## Discussion

The present study aimed to reach consensus on a set of outcomes that are considered core for the evaluation of non-pharmacological community-based health and social care interventions. The central position of this study was to use systematic, rigorous and established consensus methods to examine existing thinking and assumptions around outcomes and facilitate the voice of people living with dementia to participate in the research process through applying qualitative methodology to underpin each of the study phases and data collection approaches (including modification to Delphi survey consensus methods). Thirteen outcome items were included in the final COS (see Table 2).

### Involvement of people living with dementia in the consensus process

A key strength of this work is the involvement of people living with dementia in the research design process and as participants. The modified Delphi design reported in this study, to the best of our knowledge, is one of the first Delphi studies to be implemented successfully with people living with dementia in more than one round [27].

The reported representation of the views of people living with dementia in this study is both meaningful and substantial, and we argue without precedent in the reported literature. There has until recently been a relative lack of consultation with people living with dementia regarding the outcomes that matter most [18–21, 27]. Some have excluded the involvement of people living with dementia when ascertaining salient outcomes or not adequately reported how people living with dementia have been meaningfully facilitated to be part of the research process. While the numbers of people living with dementia who participated in this study are greater or similar to earlier and recent work, it is the process of involvement in the modified Delphi (as co-researchers designing research tools and participants) and the consensus approach that we feel sets it apart.

All consensus exercises reported in Appendix 1 in Supplementary data used workshops or discussion at key stages, but unclear reporting raises questions around whether or not the views of people living with dementia had equal weight in discussions or if people living with dementia were supported to contribute in a manner that was personally meaningful. A key issue is whether the participation and/or representation of people living with dementia is both equal and sufficient. We argue that, to optimise the responsiveness, validity and merit of COS in the field of dementia, people living with dementia should actively participate in the research process [23].

To date, this study has included 62 instances of people living with dementia being involved (17 participants in phase 1 and 18 consulted as co-researchers when designing the accessible Delphi process, 21 participated in the Delphi survey and 6 in the consensus meeting) [23, 25]. We ensured that the views and opinions of people living with dementia were given equal weight when compared with those from other stakeholder groups. This was done through the use of a modified Delphi method along with meaningful and facilitated involvement in the consensus meeting.

Participation in the online Delphi survey, which contained the same questions and wording as the modified and accessible version, was highly valued by many other participants, particularly health and social care professionals many of whom indicated in the second round that it was interesting and valuable to see how people living with dementia had rated respective outcomes. This is evident in how the scores of those from professional groups changed between rounds indicating that the views of people living with dementia had significant influence beyond their discrete participation in the study.

### The focus of the COS—‘what to measure’

The research team formed four conceptual categories (friendly neighbourhoods and home, independence, self-managing dementia symptoms and quality of life) for the purposes of structuring the Delphi survey. These categories were not the subject of any analysis or recommendations about what to measure. The wording and interpretation of all of the outcomes that were included in the Delphi survey were based on the perspectives of people living with dementia and care partners. In their capacity as co-researchers, they assisted framing the outcomes based on their primary lived experience [25]. Because of this, people with lived experience of dementia have substantially shaped, beyond their participation in a survey, the scope and focus of these 13 outcome items, and therefore what is deemed important in the context of non-pharmacological health and social care programmes.

It is important to consider the focus of the 13 core outcome items in the context of contemporary trial-related research. A recent review of 676 dementia trials and 129 mild cognitive impairment trials (311 reported non-pharmacological interventions) showed that cognitive outcomes were reported in 70% of trials, 29% measured functional performance and only 13% used quality of life measures [6]. The specific nature of the 13 core outcome items is also clearly different to existing consensus exercises where broad established domains and or existing outcome measurement instruments tend to be recommended [16–21]. Dementia is a cognitive disorder, and cognition is an outcome that is present in four of six existing consensus recommendations (see Appendix 1 in Supplementary data). Nine outcomes in the Delphi survey were cognitive (language/word finding; working with numbers; short-term memory; long-term memory; processing visual information; knowing where you are; learning new things; alertness; repeated questioning). However, only two cognitive outcomes remained in the final COS: alertness and knowing where you are. This suggests that cognitive outcomes, while having some importance, should not have a dominant focus when designing interventions and trials. It is likely that the relative lack of importance attributed to cognition in the COS reflects the extent to which the COS has been influenced by key stakeholders, including people living with dementia and less so by professional groups such as researchers and health and social care professionals.

Comparing our COS with a recent COS relating to physical activity programmes for people living with dementia shows some commonalities. There is some overlap with four of the seven outcomes: preventing falls; doing what you can do; enjoying the moment; and feeling useful and having a purpose [27]. There is also likely to be some overlap between many of the 13 core outcome items reported in this study and social health. The emergent concept of social health is based around the factors associated with preserving the autonomy and independence of people living with dementia, supporting participation in social interactions and meaningful activities [28]. This concept is particularly relevant as an outcome for people living with dementia living at home. Social health in dementia is suggested to have three key dimensions, namely: personal; disease-related; and social and physical environment influencing factors [28]. Six of the outcome items: meaningful activities; importance of relationships; communication; having a laugh; feeling valued and respected; and a sense of who you are can easily be mapped into the concept of social health and its influencing factors as set out by Dröes and INTERDEM group (a Pan-European network of researchers focusing on Early detection and timely INTERvention in DEMentia) colleagues [28]. Other outcome items could also function as proxies of areas of importance to social health (such as feeling safe and secure at home and falls), while symptom-related factors are key influencing factors across the dimensions of social health (such as alertness, losing interest, knowing where you are). Vernooij-Dassen and Jeon note that ‘the results of those interventions focusing on social health are gradually contributing to a turning point in dementia care and policy: the replacement of the disaster scenario with the scenario of living well with dementia’ [29]. However, while we suggest many of the core outcome items overlap with the concept social health, the core outcome items could also overlap with the content of existing and established domains and measurement instruments. This will be determined in the final phase of our study when we undertake a systematic review to identify relevant outcome measurement instruments, their face validity of measuring the 13 core outcome items and other measurement properties.

### Limitations

Although we successfully adapted the Delphi method to be accessible to people living with dementia, other groups only had access to an online survey, which may have not been accessible to some people in the other participant groups. The Delphi method relies on participants being open to changing their views based on being able to interpret other participant’s scores. We chose to illustrate aggregate group scores in histograms in the online survey. However, the extent with which professionals aligned their scores to the views of people living with dementia in round 2 suggests that participants in the online survey were able to interpret the histograms. This suggests that a survey-based method can be a means of reaching a consensus in the context of dementia research, although our use of a face-to-face consensus meeting also highlights the benefits of rich and detailed facilitated face-to-face discussion when seeking to attain consensus.

The consensus criteria and analytical strategy used in the Delphi survey assumed individuals participated based on their experiences of being from one of the five stakeholder groups. However, in some instances, participants did not always identify with a single stakeholder category.

Participants in the Delphi were mostly based across England, and people living with dementia were exclusively from the north-west of England. Further work may need to be undertaken to ascertain whether this COS could be applicable to interventions and trials in other countries. Furthermore, those people living with dementia who participated were representative of those with earlier or mid-stage dementia rather than those with late-stage dementia; the cognitive and communication difficulties associated with dementia would have precluded some persons with dementia from taking part.

## Conclusion

The aim of this study was to apply rigorous consensus methods to attain agreement from key stakeholders, including people living with dementia, on what outcomes should be measured as a minimum in all non-pharmacological community-based health and social care trials. Thirteen outcomes items are considered core; these are what people value in order to live well with dementia and many relate to the concept of social health. In the longer term, the use of the final COS endorsed by key research funders will help reduce inconsistent reporting of outcome data. Trialists, researchers and commissioners will then be more able to compare effectiveness across non-pharmacological community-based health and social care interventions for people with dementia living at home. Improving the quality of dementia care research evidence will help improve dementia care services within the health and social care system.

## Supplementary Material

aa-19-0731_afaa015
