# Supplementary material for: What is important to people with dementia living at home? A set of core outcome items for use in the evaluation of non-pharmacological community-based health and social care interventions
Source: Age Ageing. 2020 Feb 17;49(4):664–71. doi: 10.1093/ageing/afaa015 (PMC7331097; doi:10.1093/ageing/afaa015)
Supplement: aa-19-0731_afaa015 [file aa-19-0731_afaa015.pdf]

What is important to people with dementia living at home? A set of core outcome items for use in the evaluation of non-pharmacological community-based health and social care interventions

SUPPLEMENTARY DATA

Appendix 1 - Summary of outcome consensus recommendations[1]

Author, year of publication	Scope specification	Stakeholders involved	Consensus process	Outcomes recommended (including categories or domains and outcome measures (if reported)) concerning people living with dementia
Katona et al, 2007 [16]	Care: Defining and measuring treatment benefit in dementia	34 professionals and 2 carers	Two consensus group meetings	Cognition; Behavioural and psychological symptoms; Quality of life; Global assessments; Activities of Daily living
Moniz-Cook et al, 2008 [17]	Research: Psychosocial intervention research in dementia care	Up to 19 experts participated in the face to face consensus workshops. 131 professionals and 5 carers involved in web-based consultation.	Three face to face consensus workshops<br>A web-based pan-European consultation (email)<br>A systematic literature review	Mood (CSDD or GDS-12);<br>Patient Quality of Life (QOL-AD, DQOL, EQ-5D);<br>Patient ADL/IADL (Lawton PSMS-IADL);<br>Patient behaviour (NPI);<br>Global patient measures (GBS, CIBIC-Plus)
JPND, 2015 [18]	Research: Psychosocial intervention research in dementia care (update of Moniz-Cook et al, 2008)	Number of participants who participated in workshop 1 is not reported. However, workshop 2 involved 25 professionals. Attendees for workshop 3 also unclear, but assumed to be 25.<br>Consultation with people living with dementia was piloted with 5 people. It is reported that after the pilot the consultation involved 25 people living with dementia and 18 carers.	Three face to face consensus workshops<br><br>Consultation with people living with dementia and carers<br><br>Desk -based work	Mood (CSSD, GDS-15, RAID);<br>Quality of life (QOL-AD, DQOL; QUALIDEM; DEMQoL, QUALID); Health related quality of life (EQ-5D);<br>ADL/IADL (Lawton PSMS-IADL, Katz ADL, ADCS-ADL, BADSL, DAD)
ICHOM, 2016 [19]	Care:<br>All types and all stages of dementia	19 professionals, 3 people living with dementia and 1 carer	Literature review<br><br>Discussions with persons with dementia and patient represented groups<br><br>Workshop (participant groups unclear)	Symptoms, Functioning & Quality of Life<br>Neuropsychiatric (NPI); Cognitive (MoCA); Social (includes community affairs and relationships, but no outcome measure recommended); Daily living (BADSL); Overall Quality of Life and Wellbeing (QOL-AD & QWB-SA)<br>Sustainability: Time to full time care<br>Safety: Falls

For Review Only

				<u>Clinical status :</u> Disease progression (CDR); Hospital admissions; Overall survival

Webster et al, 2017 [20]	Care:<br>Disease modification interventions for people living with mild to moderate dementia	4 people living with dementia, 13 carers and 1 PPI member were involved in the patient and public involvement consultation and email consultation.<br><br>29 professionals participated in the consensus conference.	Systematic review<br><br>Patient and Public Involvement Consultation (focus groups, follow up email consultation and an unspecified number of interviews)<br>Consensus conference	<u>Core</u><br>Cognition (MMSE OR ADAS-Cog);<br>Biological markers (MRI)<br><br><u>Important, but not core</u><br>*Neuropsychiatric symptoms (NPI);*<br><br>*ADL (DAD);*<br><br>*Quality of Life (DEMQOL);*<br><br>*Global functioning (CDR)*
ROADMAP, 2018 [21]	Care:<br>To identify a priority set of real world dementia outcomes, across disease spectrum, from pre-clinical to severe stages	29 people living with dementia in patient and public involvement consultation.<br><br>25 people living with dementia, 70 carer's and 238 professional's participated in surveys.	Systematic review<br><br>Patient and public involvement consultation<br><br>Three discrete stakeholder surveys (for people living with dementia, carers and professionals)	Functional ability and independence;<br>Patient quality of life;<br>Behavioural and neuropsychiatric symptoms;<br>Cognitive abilities

[1] We have summarised these according to the three domains present in the Core Outcome Set-Standards for Development (COS-STAD) recommendations: scope specification, stakeholders involved and consensus process (23). We have also summarised the outcomes that these consensus exercises recommend – these tend be broad outcome domains that have sub-categories or constructs.

For Review Only

Appendix 2 - Key modification to the consensus criteria

We made post hoc modifications to the protocol in relation to the consensus criteria for inclusion of outcomes in the COS. In the original protocol we adopted what is regarded as the standard consensus approach that is widely used in the analysis of COS Delphi surveys that use a 9-point scale. As set out in the protocol:

Each outcome by stakeholder group will be classified as:

- ***Consensus in:*** *70% or more participants scoring as "very important" and less than 15% participants score as "not particularly important";*
- ***Consensus out:*** *70% or more participants scoring as "not particularly important" and less than 15% of participants scoring as "very important";*
- ***No consensus:*** *anything else not included in the other two categories.*

A list of the outcome items where "Consensus in" was met for one or more stakeholder groups were presented in the following consensus meeting.

We decided for two reasons that the 3-point scoring scale did not align to the configuration and assumptions present in the traditional consensus criteria. Firstly, the 3-point scale has fewer options for participants when compared to a 9-point scale and fewer options limit variability. Secondly, our 3-point scale was positively orientated to accommodate people living with dementia. On the other hand, the traditional consensus criteria has an assumption that some outcomes will be rated as "Not Important" or "Strongly Disagree", which is not the case with our modified and positively orientated 3-point scale. In order to reflect the positive orientation of the scale, we considered it necessary to adjust the original 'consensus out' criteria by reflecting this in the boundaries of consensus. We therefore chose to modify the 'consensus out' criteria, from:

- 70% or more of each group scored "Not particularly important" and less than 15% of participants scoring as "Very important"

to:

For Review Only

- Less than 70% of participants in each group scored “Very important”.

We did not modify the ‘consensus in’ criteria. The final consensus criteria that the study adopted was:

Each outcome by stakeholder group was classified as:

- *Consensus in: 70% or more participants in each group scoring as “very important” and less than 15% participants in each group score as “not particularly important”;*
- *Consensus out: less than 70% of participants in each group scoring as “Very important”*
- *No consensus: anything else not included in the other two categories.*

For Review Only

Appendix 3 - Ratings by each respondent stakeholder group of the importance of the 54 outcome items at Round 1 and Round 2 of the Delphi, along with the decisions made at Round 2 and the consensus meeting

							**People living with dementia**	**Carers**	**Health & Social Care Professionals**	**Policy Makers**	**Researchers**		
**Domain**	**No.**	**Outcome item**	**Lay outcome term (if applicable)**	**Lay description of outcome**	**Outcome Example**	**Delphi Round**	**Per cent of respondents rating outcome:** very important, important, not particularly important					**R2 Result (In, Out, No consensus)**	**Consensus meeting outcome**
	1	Language/word finding	NA	Finding words you want to use or say is…		R1	38, 43, 19	63, 33, 4	82, 18, 0	75, 25, 0	73, 25, 2		
**Self-Managing Dementia Symptoms**						R2	45, 50, 5	62, 38 ,0	68, 30, 2	58, 42, 0	55, 44, 2	Out	NA
	2	Working with numbers	NA	Working with numbers is…		R1	14, 48, 38	25, 53, 23	15, 65, 20	17, 67, 17	14, 67, 19		
						R2	25, 40, 35	16, 59, 25	16, 69, 16	17, 58, 25	6, 78 16	Out	NA
	3	Fear of deterioration	Dementia getting worse	Fear of dementia getting worse is…		R1	38, 24, 38	65, 23, 12	80, 18, 1	100, 0, 0	80, 20, 0	No consensus	
						R2	50, 20, 30	69, 23, 8	66, 32, 2	75, 25, 0	59, 39, 2	No consensus	Out

For Review Only

	4	Memory	Short term memory	Being able to remember things is…		R1	24, 57, 19	60, 37, 4	80, 19, 1	67, 33, 0	80, 19, 2		
						R2	50, 45, 5	54, 44, 2	53, 45, 2	58, 42, 0	49, 47, 4	Out	NA
	5	Long term memory	NA	Being able to remember things from the past is…		R1	33, 33, 33	44, 53, 4	65, 32, 2	50, 50, 0	59, 34, 7		
						R2	35, 40, 25	38, 58, 4	49, 49, 2	17, 83, 0	33, 62, 6	Out	NA
	6	Processing visual information	NA	Being able to recognise, understand and interpret objects is…		R1	38, 52, 10	65, 26, 9	73, 26, 1	75, 25, 0	64, 34, 2		
						R2	50, 45, 5	67, 33, 0	61, 38, 1	42, 58, 0	51, 49, 0	Out	NA
	7	Understanding time and place	Knowing where you are	Being able to find your way around a familiar place is…		R1	60, 25, 15	75, 23, 2	82, 18, 0	92, 8, 0	86, 12, 2		
						R2	60, 30, 10	83, 17, 0	88, 12, 0	83, 17, 0	91, 10, 0	No consensus	In
	8	Learning new things	NA	Being able to learn new things is…	For example, learning how to use a new cooker or telephone.	R1	19, 57, 24	19, 49, 32	28, 54, 18	25, 50, 25	34, 57, 9		
						R2	25, 55, 20	14.6, 50, 35	16, 67, 17	25, 43, 33	123, 82, 6	Out	NA
	9	Alertness	NA	Being aware of your surroundings indoors and outdoors is…	For example, being around hot food or drink, or crossing the road.	R1	67, 29, 5	74, 19, 7	72, 26, 1	58, 42, 0	72, 24, 3		

For Review Only

						R2	80, 20, 0	79, 17, 4	84, 16, 0	75, 25, 0	82, 18, 0	In	In
	10	Hallucinations	NA	Seeing or hearing things that aren't there is…		R1	25, 10, 65	46, 27, 27	50, 40, 10	36, 55, 9	40, 44, 16		
						R2	16, 21, 63	37, 46, 17	28, 56, 16	8, 67, 25	15, 62, 23	Out	NA
	11	Agitation	NA	Being settled and free from restlessness is…		R1	43, 38, 19	69, 25, 5	83, 17, 0	67, 33, 0	63, 34, 3		
						R2	65, 25, 10	84, 17, 0	84, 16, 0	75, 25, 0	53, 46, 2	No consensus	Out
	12	Aggression	NA	Not being aggressive is…	For example, not shouting at someone, becoming hostile or violent.	R1	62, 24, 14	67, 18, 15	68, 28, 4	58, 42, 0	57, 34, 9		
						R2	75, 20, 5	83, 15, 2	74, 26, 0	67, 33, 0	67, 27, 6	No consensus	Out
	13	Depression	NA	Few times of feeling very sad, despairing and hopeless is…		R1	25, 45, 30	71, 20, 9	79, 21, 1	58, 42, 0	73, 25, 2		
						R2	25, 60, 15	77, 19, 4	65, 34, 1	50, 50, 0	66, 31, 4	No consensus	Out
	14	Anxiety	NA	Having moments of being calm and free from troubling worries is…		R1	48, 43, 10	77, 18, 5	85, 13, 1	42, 58, 0	86, 12, 2		

For Review Only

						R2	55, 45, 0	85, 15, 0	86, 13, 0	58, 42, 0	82, 16, 2	No consensus	Out
	15	Apathy/Indifference	Losing interest	Keeping interested in things you like is…		R1	71, 29, 0	60, 32, 9	79, 21, 0	83, 17, 0	80, 20, 0		
						R2	80, 20, 0	73, 25, 2	91, 9, 0	83, 17, 0	95, 6, 0	In	In
	16	Disinhibition	NA	Acting and speaking appropriately is…	For example, not doing or saying something unsuitable.	R1	55, 35, 10	51, 40, 9	58, 38, 9	42, 42, 17	40, 47, 14		
						R2	68, 26, 5	46, 50, 4	53, 43, 4	25, 58, 17	33, 58, 9	Out	NA
	17	Appetite/Eating	NA	Having few eating or appetite difficulties is…		R1	24, 43, 33	30, 52, 18	50, 43, 7	67, 33, 0	44, 47, 8		
						R2	20, 55, 25	25, 66, 9	36, 57, 7	50, 50, 0	27, 62, 11	Out	NA
	18	Secretiveness	NA	Avoiding secrecy is…	For example, keeping important thoughts or actions from family, friends, or professionals.	R1	15, 60, 25	33, 46, 20	44, 42, 14	18, 64, 18	41, 41, 19		
						R2	15, 65, 20	26, 47, 28	29, 59, 13	9, 73, 18	15, 72, 13	Out	NA
	19	Inability to be still	NA	Ability to be still for most of the time is…	For example, not constantly pacing or wringing hands.	R1	5, 33, 62	30, 41, 30	25, 29, 46	17, 58, 25	14, 50, 36		
						R2	10, 30, 60	13, 54, 33	11, 40, 50	0, 33, 67	4, 38, 58	Out	NA
	20	Repeated questioning	NA	Not asking the same questions again and again is…		R1	38, 19, 43	36, 41, 23	21, 50, 29	25, 58, 17	30, 44, 26		

For Review Only

						R2	30, 25, 45	17, 60, 23	14, 62, 24	0, 67, 33	11, 60, 29	Out	NA
	21	Suspicion/paranoia	NA	Being free from suspicious thoughts is...		R1	24, 29, 48	55, 26, 19	63, 33, 4	58, 33, 8	46, 44, 11		
						R2	40, 25, 35	48, 41, 11	51, 43, 6	25, 67, 8	35, 51, 15	Out	NA
	22	Sleeping	NA	Being able to sleep well most of the time is...		R1	14, 48, 38	67, 19, 14	70, 27, 2	58, 33, 8	64, 34, 2		
						R2	35, 50, 15	71, 23, 6	57, 41, 2	50, 42, 8	49, 51, 0	No consensus	Out
	23	Frustration	NA	Being free from frustration most of the time is...		R1	45, 40, 15	75, 20, 5	80, 19, 1	50, 42, 8	73, 22, 5		
						R2	60, 25, 15	77, 23, 0	76, 24, 0	58, 42, 0	69, 29, 2	No consensus	Out
	24	Nightime behaviour	NA	Being settled at night time is...		R1	33, 33, 33	75, 14, 11	69, 30, 1	42, 58, 0	54, 39, 7		
						R2	55, 20, 25	79, 15, 6	64, 35, 1	50, 50, 0	47, 47, 6	No consensus	Out
**Quality of Life**	25	Physical function	NA	Being physically able to carry out everyday activities without difficulty is...		R1	48, 29, 24	56, 31, 13	61, 38, 1	33, 58, 8	60, 38, 2		
						R2	60, 25, 15	58, 38, 4	63, 37, 0	33, 58, 8	62, 38, 0	Out	NA

For Review Only

	26	Mobility	NA	Being able to walk or get around without too much difficulty is…		R1	57, 33, 10	63, 26, 11	65, 35, 0	33, 67, 0	67, 32, 3		
						R2	75, 15, 10	69, 29, 2	71, 30, 0	33, 67, 0	76, 24, 0	No consensus	Out
	27	Activeness	Keeping physically active	Taking part in as much physical activity as you would like is…		R1	43, 43, 14	48, 39, 13	57, 37, 6	42, 58, 0	56, 39, 5		
						R2	45, 50, 5	52, 44, 4	51, 48, 1	50, 50, 0	47, 50, 2	Out	NA
	28	Stability	Falls	Not falling at home or when out and about is…		R1	50, 30, 20	87, 4, 9	89, 11, 0	75, 25, 0	91, 9, 0		
						R2	79, 16, 5	92, 4, 4	92, 8, 0	83, 17, 0	93, 7, 0	In	In
	29	Hygience & comfort	Personal hygiene & cleanliness	Being as clean and comfortable as you would like is…		R1	71, 29, 0	77, 13, 9	78, 19, 2	75, 25, 0	81, 17, 2		
						R2	80, 20, 0	92, 4, 4	92, 8, 0	100, 0, 0	91, 9, 0	In	In
	30	Vision & hearing	NA	Being able to see, hear and understand is…	For example, being able to see, hear and understand people you are talking to.	R1	57, 33, 10	73, 35, 2	83, 17, 1	75, 25, 0	81, 19, 0		
						R2	70, 25, 5	81, 17, 2	85, 15, 0	75, 25, 0	86, 15, 0	In	In
	31	Health conditions	NA	Having as few other illnesses as possible is…		R1	52, 38, 10	67, 25, 7	73, 27, 1	42, 58, 0	62, 38, 0		

For Review Only

						R2	65, 30, 5	71, 27, 2	77, 23, 0	50, 50, 0	56, 44, 0	No consensus	Out
	32	Happy	NA	Having moments of feeling happy and relaxed is…		R1	67, 24, 10	85, 13, 2	92, 8, 0	75, 25, 0	86, 14, 0		
						R2	65, 30, 5	89, 11, 0	95, 5, 0	75, 25, 0	94, 6, 0	No consensus	Out
	33	Having a laugh	NA	Feeling able to have a laugh with other people is…		R1	57, 38, 5	80, 19, 2	84, 16, 0	58, 42, 0	81, 17, 2		
						R2	70, 30, 0	79, 19, 2	84, 16, 0	58, 42, 0	89, 9, 2	No consensus	In
	34	A sense of who you are	NA	Feeling able to keep your identity is…		R1	67, 14, 19	91, 7, 2	89, 10, 1	82, 18, 0	97, 3, 0		
						R2	70, 20, 10	85, 15, 0	91, 9, 0	91, 9, 0	98, 2, 0	In	In
	35	Having a sense of purpose, role & occupation	NA	Feeling you have a sense of purpose in your life is…		R1	62, 38, 0	62, 35, 4	85, 14, 1	67, 33, 0	88, 12, 0		
						R2	65, 35, 0	68.8, 29.2, 2.1	87.6, 12.4, 0	75, 25, 0	89.1, 10.9, 0	No consensus	Out
	36	Self-esteem, self-efficacy & confidence	Self-esteem & confidence	Having moments of good self-esteem and feeling confident is…		R1	52, 43, 5	74, 24, 2	83, 17, 0	50, 50, 0	88, 12, 0		

For Review Only

						R2	55, 45, 0	77, 23, 0	81, 19, 0	50, 50, 0	87, 13, 0	No consensus	Out
	37	General health	NA	Feeling generally healthy while having a dementia diagnosis is…		R1	52, 43, 5	72, 24, 4	81, 18, 1	42, 58, 0	84, 16, 0		
						R2	55, 40, 5	77, 23, 0	78, 22, 0	67, 33, 0	80, 20, 0	No consensus	Out
	38	Acceptance of Dementia	NA	Accepting changes in your life that have come with dementia is…	For example, accepting changes in relationships and carrying out everyday activities.	R1	42, 53, 5	59, 33, 7	71, 28, 1	33, 67, 0	66, 34, 0		
						R2	53, 42, 5	58, 38, 4	65, 35, 0	8, 83, 8	58, 42, 0	Out	NA
	39	Feeling like a burden	NA	Having moments of not feeling a burden is…		R1	48, 38, 14	67, 26, 7	84, 16, 0	58, 42, 0	77, 21, 2		
						R2	55, 30, 15	63, 33, 4	77, 23, 0	50, 50, 0	73, 27, 0	No consensus	Out
	40	Embarrassment	NA	Not feeling too embarrassed is…	For example, of having dementia or if you have difficulty thinking of a word or forgetting someone's name.	R1	15, 20, 65	57, 28, 15	81, 18, 1	50, 50, 0	71, 28, 2		
						R2	30, 30, 40	50, 37, 13	51, 41, 9	25, 67, 8	36, 51, 13	Out	NA

For Review Only

	41	Loneliness	NA	Not feeling lonely or isolated is…		R1	43, 33, 24	81, 15, 4	92, 8, 0	92, 8, 0	90, 9, 2		
						R2	53, 37, 11	81, 15, 4	85, 15, 0	75, 25, 0	91, 7, 2	No consensus	Out
	42	Feeling financially secure	Financial security	Feeling financially secure and comfortable with financial arrangements is…		R1	48, 38, 14	65, 30, 6	70, 29, 2	64, 36, 0	60, 36, 3		
						R2	50, 35, 15	59.6, 38.3, 2.1	62.9, 37.1, 0	50, 50, 0	54.6, 43.6, 1.8	Out	NA
**Friendly Neighbourhood & Home**	43	Communication	NA	Being able to communicate with others is…	For example, being able to hold a conversation or reply to a letter or email.	R1	81, 14, 5	77, 21, 2	87, 13, 0	75, 25, 0	86, 14, 0		
						R2	85, 15, 0	90, 8, 2	93, 7, 0	92, 8, 0	98, 2, 0	In	In
	44	Social contact/company	NA	Having company or social contact if you want it is…	For example, this could mean having someone who wants to listen.	R1	33, 57, 10	81, 11, 7	87, 13, 0	75, 25, 0	90, 9, 2		
						R2	55, 45, 0	79, 17, 4	81, 19, 0	67, 33, 0	78, 22, 0	No consensus	Out

For Review Only

	45	Having a sense of social integration	NA	Feeling included in your neighbourhood or community is…	For example, attending or taking part in neighbourhood or community groups or events if you want to.	R1	19, 52, 29	43, 37, 20	58, 37, 5	42, 50, 8	57, 40, 3		
						R2	25, 50, 25	27, 56, 17	40, 52, 8	42, 50, 8	40, 56, 4	Out	NA
	46	Importance of relationships	NA	Continuing good relationships with people who are important to you is…	For example, family close friends, and other people important to you.	R1	85, 15, 0	89, 8, 4	93, 7, 0	92, 8, 0	93, 7, 0		
						R2	95, 5, 0	90, 8, 2	99, 1, 0	100, 0, 0	98, 2, 0	In	In
	47	Feeling that the neighbourhood is safe & secure	Feeling that the outdoors is safe & secure	Feeling safe and secure when outdoors is…		R1	45, 30, 25	80, 17, 4	85, 15, 0	75, 25, 0	81, 19, 0		
						R2	60, 25, 15	77, 21, 2	79, 21, 0	67, 33, 0	76, 24, 0	No consensus	Out
	48	Feeling safe and secure	NA	Feeling safe and secure at home is…		R1	43, 43, 14	91, 7, 2	94, 6, 0	92, 8, 0	93, 7, 0		
						R2	70, 25, 5	94, 6, 0	93, 7, 0	83, 17, 0	91, 9, 0	In	In

For Review Only

	49	Feeling valued and respected by others	NA	Feeling valued and respected by others is…	For example, friends, family, professionals and people in the community.	R1	48, 48, 5	80, 19, 2	80, 20, 0	42, 58, 0	81, 17, 2		
						R2	50, 45, 5	75, 21, 4	79, 21, 0	42, 58, 0	75, 24, 2	No consensus	In
	50	Reaction of family/friends to diagnosis	NA	Knowing that your family and friends accept and understand you with your diagnosis is…		R1	45, 55, 0	79, 13, 8	89, 11, 1	50, 50, 0	88, 12, 0		
						R2	63, 37, 0	83, 15, 2	81, 19, 0	67, 33, 0	82, 18, 0	No consensus	Out
	51	Carer reaction	NA	The reaction of your carer or care partner to the things you do or say as a result of dementia is…	For example, your care partner could be a family member or another significant person or friend. Things might include forgetfulness or behaving differently than you used to.	R1	68, 32, 0	82, 9, 9	92, 8, 1	58, 42, 0	84, 14, 2		
						R2	79, 21, 0	87, 11, 2	92, 8, 0	58, 42, 0	89, 9, 2	No consensus	Out
**Independence**	52	Daily activities (basic)	NA	Being able to carry out everyday tasks is…	For example, eating, bathing and dressing yourself is…	R1	67, 24, 10	50, 38, 12	71, 26, 2	50, 50, 0	71, 28, 2		
						R2	70, 25, 5	50, 48, 2	71, 28, 2	58, 42, 0	73, 27, 0	No consensus	Out

For Review Only

	53	Daily activities (advanced)	NA	Being able to carry out more complicated activities is…	For example, cooking, shopping, and managing money or medications is…	R1	43, 33, 24	25, 40, 35	27, 61, 13	17, 67, 17	30, 58, 12		
						R2	40, 50, 10	13, 53, 34	17, 69, 14	17, 42, 42	19, 76, 6	Out	NA
	54	Meaningful activities	NA	Being able to do things that you enjoy and want to keep doing is…		R1	86, 14, 0	75, 19, 6	89, 11, 0	75, 25, 0	90, 10, 0		
						R2	90, 10, 0	79, 19, 2	95, 5, 0	75, 25, 0	98, 2, 0	In	In

For Review Only
